# Accounting for Location Error in Kalman Filters: Integrating Animal Borne Sensor Data into Assimilation Schemes

**DOI:** 10.1371/journal.pone.0042093

**Published:** 2012-08-10

**Authors:** Aritra Sengupta, Scott D. Foster, Toby A. Patterson, Mark Bravington

**Affiliations:** 1 Department of Statistics, The Ohio State University, Columbus, Ohio, United States of America; 2 CSIRO Mathematical and Information Sciences, Hobart, Tasmania, Australia; 3 CSIRO Wealth from Oceans Research Flagship, Castray Esplanade, Hobart, Tasmania, Australia; National Oceanic and Atmospheric Administration/National Marine Fisheries Service/Southwest Fisheries Science Center, United States of America

## Abstract

Data assimilation is a crucial aspect of modern oceanography. It allows the future forecasting and backward smoothing of ocean state from the noisy observations. Statistical methods are employed to perform these tasks and are often based on or related to the Kalman filter. Typically Kalman filters assumes that the locations associated with observations are known with certainty. This is reasonable for typical oceanographic measurement methods. Recently, however an alternative and abundant source of data comes from the deployment of ocean sensors on marine animals. This source of data has some attractive properties: unlike traditional oceanographic collection platforms, it is relatively cheap to collect, plentiful, has multiple scientific uses and users, and samples areas of the ocean that are often difficult of costly to sample. However, inherent uncertainty in the location of the observations is a barrier to full utilisation of animal-borne sensor data in data-assimilation schemes. In this article we examine this issue and suggest a simple approximation to explicitly incorporate the location uncertainty, while staying in the scope of Kalman-filter-like methods. The approximation stems from a Taylor-series approximation to elements of the updating equation.

## Introduction

The process of updating physical ocean models using observations, to obtain accurate estimates of ocean state is referred to as data assimilation (DA) and is used to forecast current and future ocean conditions, as well as for hind-casting (backward smoothing) of historical states (e.g., [1], [2]). Data assimilation schemes must be computationally cheap, as the scale of oceanographic and atmospheric systems are generally large, typically with fine granularity in time, and large number of spatial cells. The ocean and atmosphere are continuously changing, so it is desirable to efficiently update model predictions (forecasts and hind-casts) with new data as it comes online. Also, the DA scheme needs to be able to use a wide variety of different data sources. Many examples of atmospheric and oceanographic models exist (for more details on data assimilation schemes see [1] and [3]). In this study we consider the use of spatially imprecise measurements in DA schemes – accurate measurements on the observed state variables, with imprecise spatial locations.

Modern biologging technology has brought a glut of observations of ocean temperature and salinity at depth (e.g., [4]), but a significant barrier to uptake of such data in physical models is the issue of spatial uncertainty. Traditionally, most data used in DA schemes are obtained from specially designed sampling devices, such as Argos floats (http://www.argo.ucsd.edu/), ship-based instruments, Lagrangian drifters and remote sensing data. These platforms provide highly accurate information but are costly to deploy. The Lagrangian drifters have been used for tracking upper ocean water circulation and sea surface temperature (e.g., [5], [6]). However, these sampling platforms tend to under-sample in some areas; for example, Argo floats are often advected away from coastal areas, or are blocked from ice prone areas (e.g., [7], [8]). In contrast, a recent and cost effective addition to the suite of ocean sampling platforms has been miniaturized instruments attached to marine animals; the sensors sample depth/pressure, ambient temperature and conductivity (e.g. [9]). Sampling rates vary between instruments, but recent monitoring devices can collect data at 1 Hz, although operational sampling rates for lengthy deployments are often as low as 1/60 Hz.

Using free-swimming animals as data collection sources has many advantages. However, the key drawback with using this source of data is that the precise location of the observations are sometimes poorly known (e.g., [10]). Location data from oceanographic drifters and Argos floats, being derived from similar observation technology, also suffer from the problems of spatial error. However, for these technologies, there is enough spatial data associated with each ocean-state measurement so that the locations which are deemed inaccurate can be discarded, or straightforwardly corrected. With data collected from animal-borne sensors this is typically not the case, and its use in DA must take this into account. This article demonstrates how incorporation of this sort of data into DA schemes is feasible, despite the associated location uncertainty.

Broadly speaking, there are two types of widely used animal-borne sensors, known as ‘tags’ : 1) data-storage (also called archival tags, see [11], [12]) for which non-spatial sensor data is used to spatially locate the animal (see [10], [13]–[14]) and, 2) satellite tags which are spatially located by satellite providers such as CLS-Argos (see www.cls.fr).

Satellite tags are attached externally to animals which spend sufficient time at the surface for the tag antenna to be exposed, and thus able to transmit. Therefore, satellite tags send near-real time data. Satellite tags are often more expensive, but have the advantage of far lower degrees of spatial error in positions (e.g., [15] and [16]). However, most satellite tags need to drastically summarize raw sensor data streams because of the low bandwidth and limited battery life available for data transmission (e.g., [17]). In the context of a DA scheme, this means that data from satellite tags could be used for forward- and hind-casting.

**Figure 1:**
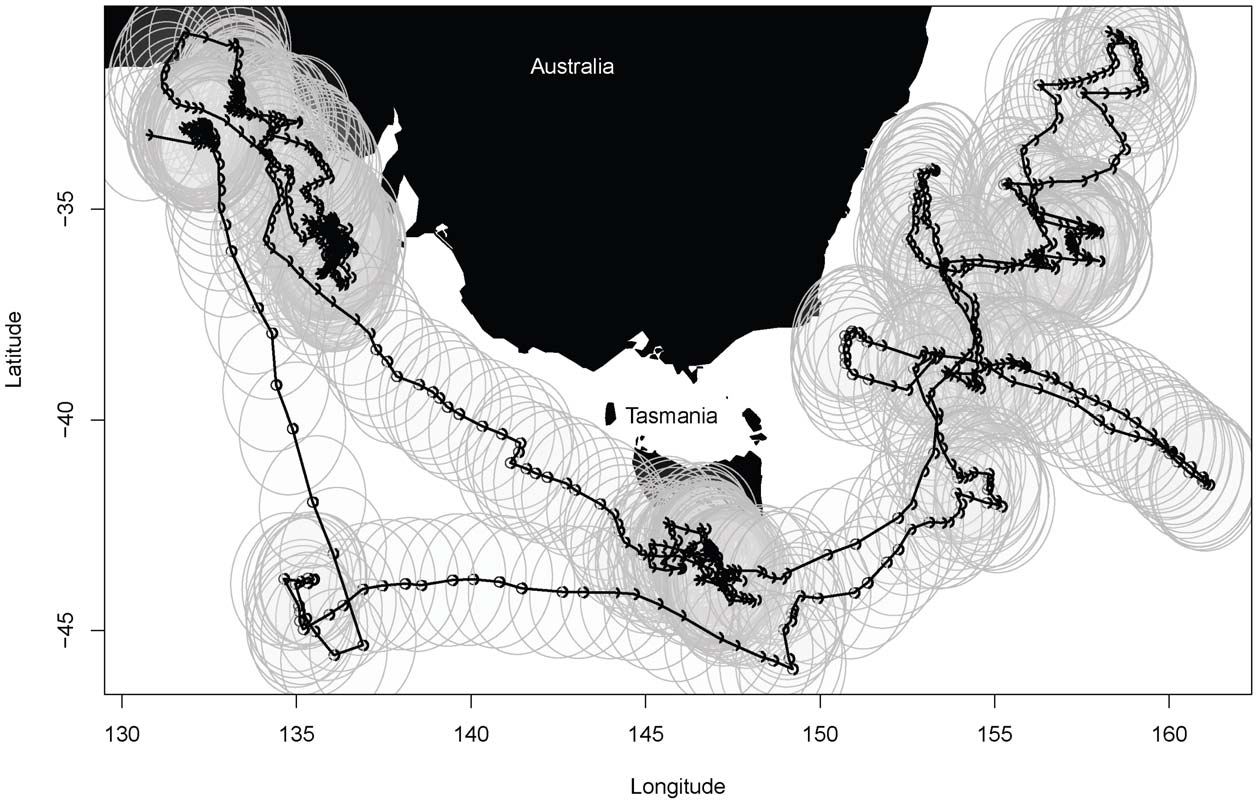
An example of movement data obtained from a data storage tag deployed on a tuna (CSIRO unpublished data). The Location estimates were derived from the state-space model approach developed by [25]. This method yields a point-estimate along with error-variances from the 95% error ellipses depicted here were derived. Associated with this track are records of temperature-at-depth recorded every minute over a period of approximately 12 months (data not shown).

In contrast, data-storage tags, typically used for tracking non-air breathing animals, simply store data on board, and data retrieval relies on the tags being retrieved when animals are recaptured. Such data are highly detailed with many thousands of observations, but are geo-located only infrequently (e.g., only twice per 24 h) and with low spatial accuracy (see example in Figure 1). In these data, latitudinal errors are typically much greater than longitudinal errors, and vary systematically through time (e.g., [18]). In the DA scheme context, these data sources are therefore retrospective, and thus are primarily useful for hind-casting.

Data assimilation for many oceanographic models is based on variants of the Kalman filter algorithm (see [19] for a statistical context), with hind-casting naturally performed by corresponding variants of Kalman smoothing (e.g., [20]). In this article we describe some simple adjustments to the Kalman filter algorithm which allow the use of spatially imprecise data in the DA scheme. We then briefly consider the applicability of these adjustments to more complex DA schemes. Note that in this article, a direct and efficient approach that could be implemented within the extensive existing libraries of oceanographic modelling code, is the key, and thus we avoid consideration of Bayesian hierarchical models which tend to be computationally prohibitive in operational DA schemes.

We proceed as follows: A brief review of the Kalman filter models and the updating equations is given. Then we detail adjustments to the Kalman filter procedure which account for location errors. We also discuss the Kalman smoothing algorithm in the presence of location errors. The setup and the results of some simple simulation studies are discussed. Finally, we discuss our methods, the scope and limitations of this study, and some of the possible extension of the results. Throughout this article we will assume that the location error variance is known for all time points although, in practice these need to be estimated. However, they may be directly obtained from dedicated studies (e.g., [21]).

In this short article, our goal is not to present a fully operational oceanographic DA scheme that handles location error; that would be a much larger task. Our contribution is to demonstrate, via application to simulated data, that accounting for spatial uncertainty is a surmountable problem, without wholesale adjustment to standard techniques.

## Materials and Methods

### The Kalman Filter

A brief review of the Kalman filter is now given. For a more thorough treatment see [19] or [22]. We use the notation used in [19] but acknowledge that this is not the only choice. A notable alternative is [23]. In Appendix S1 we give a glossary of terms and a description of each one, for both sets of terminologies. This should aid translation for those familiar with [23].

Let 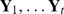 denote the observed values of a variable of interest at time points 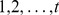 respectively. The observations may be vector or scalar depending on the particular system under study in the DA setup, and the spatial locations of these observations are given by 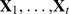. We assume that 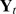 depends on an unobserved quantity 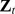, known as the state of the nature, or the system state. Typically, the value of 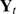 also depends on the location of the observation, 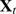. The model for 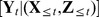, which is called the *observation equation*, is
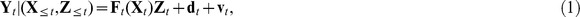
where 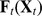 is a known quantity which may change with time and measurement location. The model for 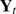 may be governed by its correlation with the components of 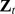. In such cases, the elements of 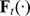 will depend on the covariance between 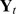 and 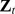. In (1), 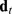 is a known vector of the same dimension as 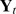 and it may, or may not change with time. The *observation error*, 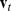, is assumed to be normally distributed with mean zero and a known variance 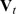, which may also be time dependent, i.e., 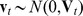.

The model for the *state of nature*, 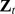, is given by the *system equation* which is of the form
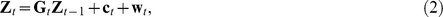
where 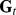 is a known quantity, 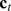 is some known vector of the same dimension as 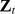, and the *system equation error*, 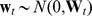, where 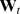 is assumed known. Here also 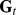, 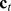 and 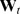 may or may not change with time. The system equation (2) does not depend on the location of 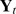. If the 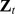 vectors are measured over space, their locations are pre-determined, and not subject to any measurement error. We also assume that 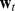 and 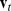 are independent.

One can write down the joint distribution 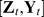, conditional on 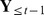 and 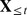 as:
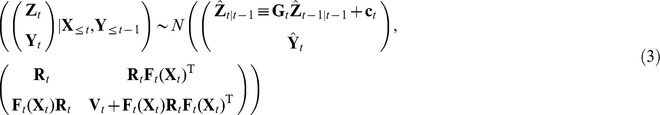
where 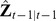 is the estimate of the system state at time point 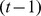 using all available information upto time 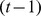, 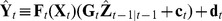, and 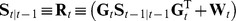 (see [19]).

Once we obtain an observation on 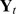, we can use the joint distribution (3) to obtain the expectation and variance for the posterior distribution 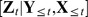. The expected value and the variance of the posterior distribution are:
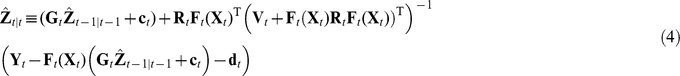
and,
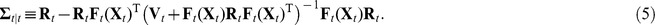
Note that the posterior distribution depends on the location 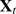.

### Adjustments for Location Error

Suppose we have an estimate for the location of an observation 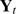, available from auxiliary data (i.e. estimates of location derived from the tag data). Let us denote the estimate by 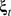. We assume that 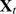, the true location of the observation, is a random variable, the distribution of which is centered around the estimate 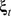 and has some variance which will be denoted by 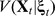. That is, we have
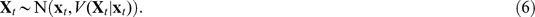



We will assume that we have an estimate 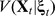 through previous experimental data (e.g. [15]). We further assume that 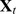 is independent between time steps. The necessary adjustments to the filtering algorithm will now be made using a first-order Taylor-series expansion.

### Adjustments to the Joint Distribution. 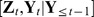


Using a first-order approximation, the expectation of 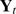 is (see Appendix S2 for all the derivations in this section)
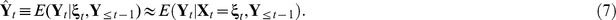
where the conditioning on the left hand side is with respect to (w.r.t.) the noisy location, and the conditioning on the right hand side is done w.r.t. the true location, treating the noisy estimate as the truth.

This suggests that using the noisy estimate of location is a valid approximation. However, there is potentially substantial bias in the variance as
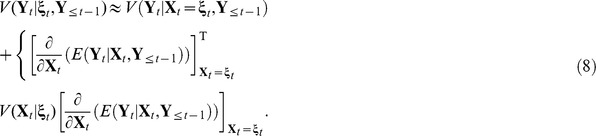
Reasuringly, the approximate variance in the unknown location situation is inflated as compared to knowing locations.

**Figure 2:**
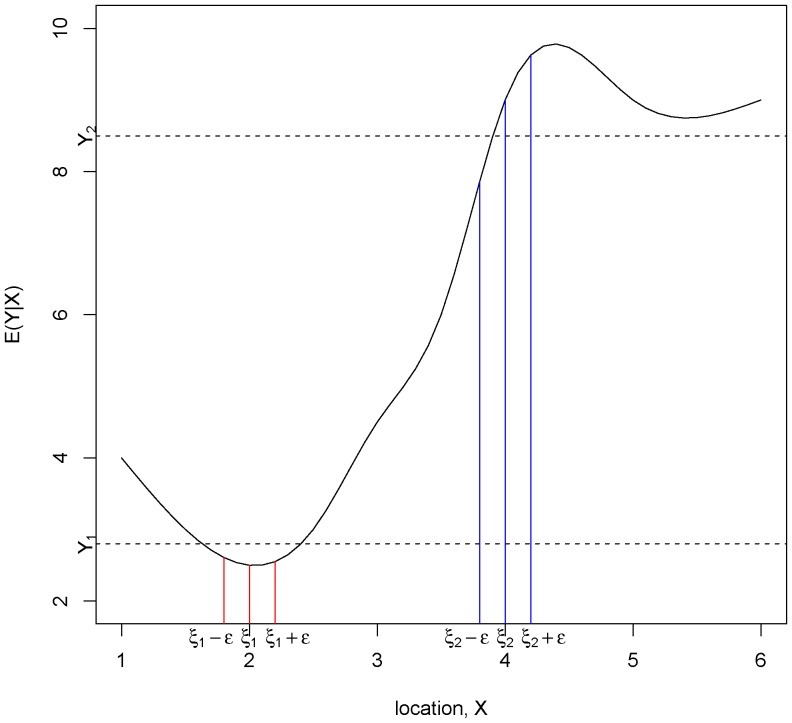
A diagrammatic representation of the problem where the curve corresponds to 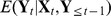
 **.** 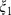 is a point with small local slope (in this case, the precision of the location is less influential) and 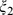 is a point with a larger local slope (here, the precise location is influential).

The term 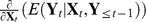 measures how much the expectation will change for a small change in the location 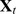. The variance inflation term in (8) will not be too significant if the local slope of the surface of the expectation around 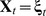 is small (see Figure 2). In such a situation, the precise location of the observation is less influential. However, if the local slope is large, then the precise location does matter. See Figure 2 for a pictorial illustration.

We complete the adjustments to the joint distribution by considering the covariance between 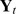 and 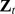,
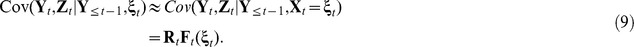
So, the covariance, like the expectation, requires no adjustment.

Combining the expectation, the variance, and the covariance, the joint distribution of 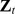 and 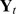, conditional on 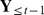 and 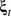 can be written as,
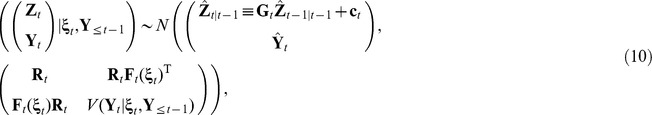
where 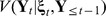 is given by (8) and 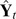 is given by (7). This should be contrasted with (3). The variance of 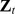 is larger than that in (3).

The Taylor-series expansion shown above retained terms up to the first-order. Better approximations may be obtained considering the higher order terms. In Appendix S3 we derive the second-order correction for the expectation. However, computation of these extra terms is more expensive and difficult to code, so we do not pursue it further in this article.

#### The Posterior Estimates

Recall that the posterior estimate for the state of the system at time point 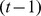 is 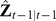, which is obtained using all the information available up to time point 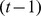. Then we can write down the joint distribution of 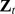 and 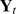, conditional on 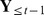 and 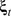, as in (10). From this joint normal distribution, we can derive the posterior distribution 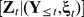 (see [20]).

The mean of the posterior distribution is
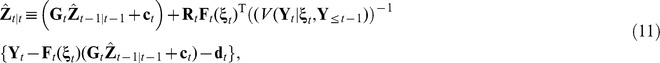
and the variance is
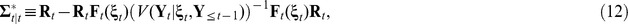
where 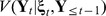 is given by (8). The posterior expectation and variance are both affected by location error through the alteration of 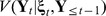. This posterior distribution gives our state of knowledge about 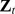 at time 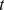, using all the information available up to that time.

### Kalman Smoothing

Until now, we have considered only Kalman filtering, where an estimate of a signal 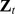 was made from considering all the previous observations. The process will produce the *best forecasts* but it will not produce the *best hindcast* (estimate of the entire time series). To obtain the best hindcasts we need to consider all the data, both previous and future. We denote this estimate as 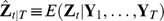 and refer to it as the Kalman smoothed estimate. We note that for many tag types (e.g. archival tags) the data from animal tags can only be used for hind-casting as the data cannot be made available in time for forecasting. This is due to the problems of tag and data retrieval, and data manipulation. It is possible that this process can be sped up in future.

A detailed discussion and the derivation of the Kalman smoothing equations, in the general setting, can be found in [20]. In our current work we are concerned with the so-called fixed-interval smoothing because of its relevance to hind-casting problems. Accounting for location error in the Kalman smoothing algorithm can be obtained directly by following the steps in [20], using the equations in the previous section.

The approach is to run a Kalman filter forward in time over the full interval 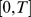, storing state estimates at each update, and then these stored quantities are run backwards in time to obtain the smoothed estimates. This process generates the smoothed estimates in reverse sequence, 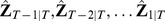. The adjusted Kalman smoothing equations are
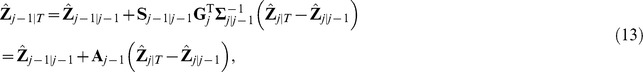
where 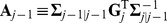. The recursion for the error covariance is
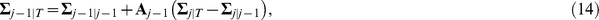
where recall that 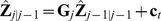, 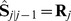, and the estimates 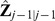 and 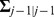 are obtained using (11) and (12).

Thus, to account for location error in Kalman smoothing one needs to only adjust the forward moving filtering process. The fixed-interval smoothing equations have no further dependence on the location of the observation. The information in the observations has already been fully incorporated during the filtering process.

## Results

### One-Dimensional Simulation System

**Figure 3:**
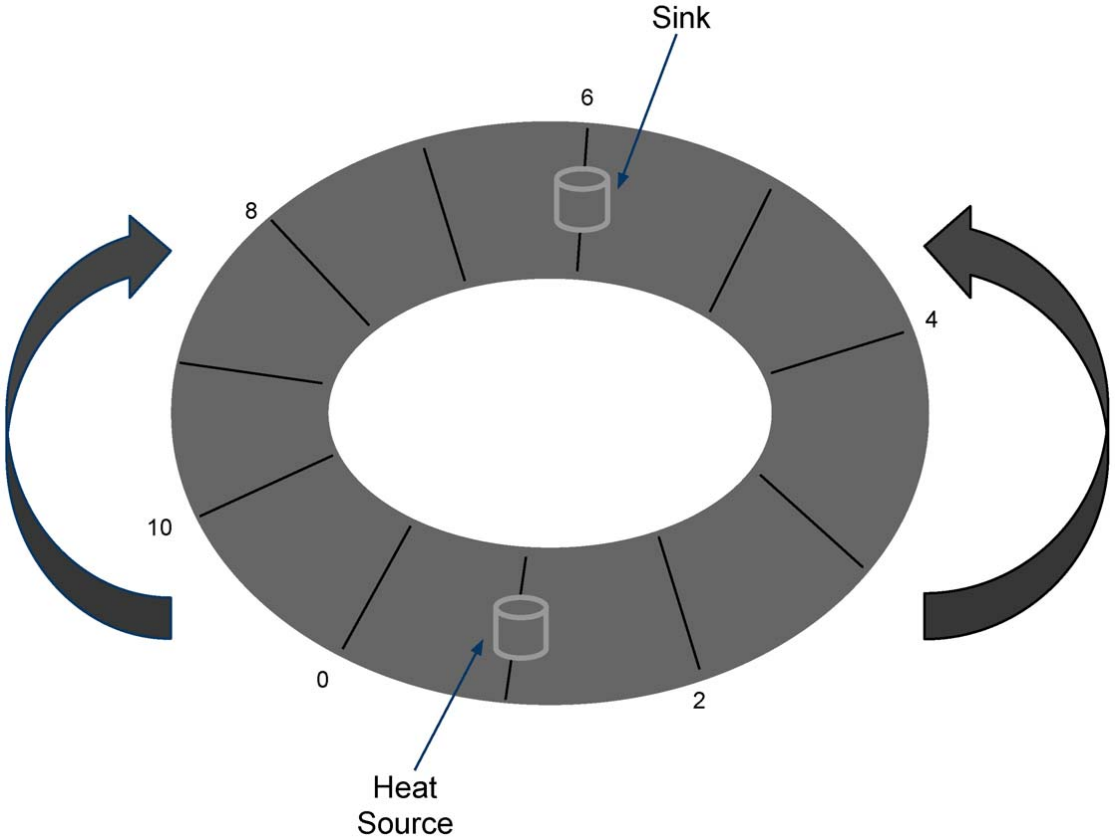
Figure showing the simulation set-up. Heat flows in the direction from the *Source* to the *Sink*.

To test the efficacy of the adjustments for location error in Kalman filter and Kalman smoothing we performed a simple one dimensional simulation along the surface of a ring. In this simple simulation, we assume that our simulated animal moves along the surface of the ring taking measurements 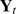 at time 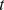 from 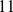 possible observation locations labelled from 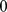 to 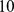 (the setup of the simulation is shown in Figure 3). Let us pretend, for the sake of illustration, that this measurement is of temperature. We introduce a temperature gradient via *a source* and *a sink* at two particular locations as shown in Figure 3. At the source, for each time point, there is an increase of 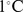 in temperature and the sink absorbs 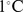. The state vector of interest is therefore 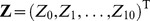, the true water temperature at each of the locations.

A continuous temperature gradient exists along the surface of the ring. However, the measured temperature of water at any given location was considered to be a linear interpolation of the two neighboring values of 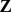, plus some random noise. The model for 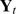 at any given time 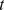, given the location of the observation 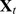, is given by
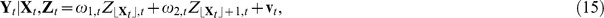
where 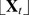 denotes the largest integer less than 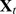 and the weights 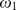 and 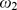 are determined as 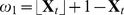 and 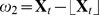. The expectation is a linear function of the true location 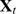. Note that 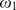 and 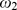 are not differentiable at the boundaries (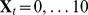). However,
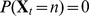
where, 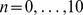. Therefore, this should not be a problem. The observation error 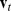 was taken to be a 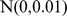 variable.

The model for the evolution of 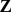 over time was taken to be
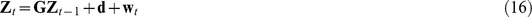
with
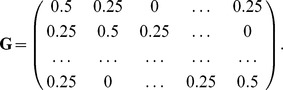
and therefore represents a diffusion process. The constant 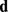 is the vector given by:
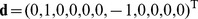
encapsulating the source and sink concept described earlier. The system equation error is 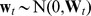, where 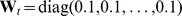.

The filtering process for each data set was initialized with all state variables equal to 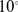
C. The system was evolved over time according to equation (16). The animal's location 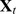 was initialized to be at location 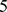 and evolves according a random walk, 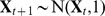. The 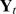 vectors were simulated according to the model given by (15), for 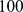 time steps. A total of 1000 data sets were created using this model.

We compared several different variants of the Kalman filtering DA scheme. We estimated the state variables using the true locations and the noisy locations. Additionally, when using the noisy locations we used the filtering process ignoring the uncertainty in locations (eqn. (3)–(5)) and also the filtering process accounting for location uncertainty (eqn. (10)–(12)). Kalman smoothing was used to hind-cast the system states. The simulated values and the predictions from the filtering and smoothing, along with the mean square prediction errors (MSPEs), were recorded from 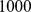 simulation runs for each of the DA schemes. This was repeated for three different values of the location-error variance, namely 0.01, 0.1, and 1.

### One Dimensional Simulation Results

A summary of MSPEs for the different methods are given in Table 1. When the measurement error variance was small 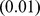, there was little difference in the results for the three procedures. This is not surprising as the locations have low uncertainty. Increasing the location error variance decreases the performance of the standard Kalman filter. In these cases the location error adjusted Kalman filter updates performs substantially better (see Table 1).

**Table 1:** Mean MSPE (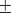 standard deviation) of a 1000 simulated data sets with predictions from Kalman filtering and Kalman smoothing.

	Location Error?	Measurement error variance		
		0.01	0.1	1
Usual KF	No	0.237 ( 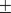 0.014)	0.238 ( 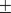 0.014)	0.237 ( 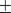 0.014)
Smoothing	No	0.185 ( 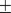 0.009)	0.186 ( 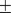 0.009)	0.185 ( 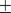 0.009)
Usual KF	Yes	0.255 ( 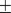 0.016)	0.442 ( 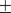 0.032)	3.515 ( 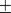 0.320)
Smoothing	Yes	0.202 ( 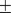 0.010)	0.392 ( 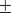 0.032)	3.892 ( 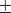 0.396)
Adjusted KF	Yes	0.251 ( 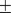 0.016)	0.313 ( 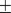 0.023)	0.538 ( 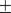 0.078)
Smoothing	Yes	0.198 ( 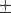 0.010)	0.253 ( 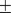 0.017)	0.423 ( 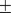 0.061)

Each data set contains 100 time steps and imitates an animal's movement over a one-dimensional ring. Location error is included and excluded (first rows) to gauge its effect.

Surprisingly, the Kalman smoothing algorithm applied to the data with noisy locations sometimes achieved a worse MSPE than the comparable Kalman filtering algorithm, when we didn't account for the noise while moving forward in time (i.e., when we used the standard Kalman filter algorithm in the presence of location errors). This is contrary to prior expectation from theoretical considerations. It implies that the underlying statistical model used in Kalman filtering does not represent the data adequately in this situation. On the other hand, applying the smoothing algorithm to the estimates obtained using the adjusted Kalman filter algorithm led to a slight improvement in the results after smoothing. This is expected and indicates that the underlying statistical model is more consistent than that which ignores the location errors.

### Two-Dimensional Simulation on the Surface of a Torus

The one-dimensional simulation was extended to two dimensions by assuming a model spatial domain where both the X-coordinates and the Y-coordinates are joined at the ends to form a torus like structure. The X-axis coordinates ranged from 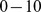 and those along the Y-axis ranged from 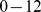. Simulations were run for 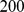 time steps and a heat source was located along the Y-coordinate 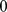 while a heat sink was located along the Y-coordinate 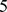.

For the two dimensional case, the observation equation considered was of the form
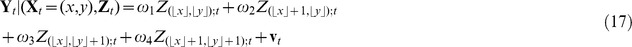
where 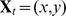 denotes the true location of the measurement. The weights 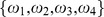 were determined according to bivariate linear interpolation (see [24]). The observation error 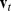 was taken to be a 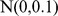 variable. The system equation to describe the evolution of the state over time was defined as
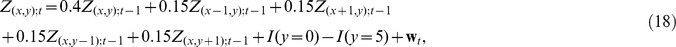
where 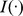 denotes the indicator function. The two terms involving the indicator function are the heat source and the sink. In these simulations the system equation error 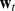 was taken to be 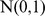. The location error variance was held at 1. A total of 1000 data sets were generated using this model and MSPE was used as the model's performance measure.

For the two dimensional simulation we again estimated the state vector using knowledge of both the true locations and the noisy locations. When using the noisy locations, we used both the usual filtering algorithm and the location error adjusted algorithm. As in the one-dimensional study, we applied both the filtering and the smoothing phase, again repeating the simulation/estimation procedure for 1000 iterations, setting the location error variance to one. A summary of the results are shown in Table 2.

**Table 2:** Mean MSPE (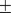 standard deviation) of a 1000 simulated dat sets with predictions from Kalman filtering and Kalman smoothing.

	MSPE ( 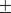 S.D)
Usual KF updates; no location error	1.98 ( 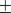 0.12)
Smoothing	1.85 ( 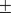 0.08)
Usual KF updates in presence of location errors	3.86 ( 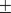 0.71)
Smoothing	4.28 ( 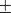 0.94)
Adjusted KF updates	2.27 ( 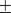 0.25)
Smoothing	2.18 ( 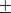 0.21)

Each data set contains 200 time steps and imitates an animal's movement over a torus. Location error is included and excluded (first rows) to gauge its effect.

In essence, the two-dimensional simulation results were concordant with the one dimensional simulation results. The adjusted Kalman filter updating equations again gave results that were better than those from an unadjusted DA scheme when there was uncertainty in location. In the presence of location error, the smoothing algorithm applied to the estimates obtained by applying the usual filtering process, produce estimates with larger MSPEs than those obtained from the filtering process, again indicating that the underlying statistical model is no longer valid. When we applied smoothing to the estimates obtained by applying the location error adjusted updating equations, there was again an improvement in the MSPEs. In summary, the two dimensional simulation results suggest that the adjusted updating equations performs better than the usual updating equations, when errors affect the measured location of system observations. This is in complete agreement with the one dimensional simulations.

## Discussion

Our motivation for this study was the challenge of utilizing the voluminous amounts of sensor data collected from marine animals for oceanographic data assimilation schemes. In this article we demonstrated how to make simple adjustments to a standard Kalman filtering DA scheme to account for the uncertainty in the spatial location of the observations. Our simulations demonstrated that that the adjustments were effective and gave better results than a standard Kalman filtering DA scheme, given error prone location estimates. While our example is simple, we note that our adjustment method can also be extended to non-linear filtering schemes like the extended Kalman filter (EKF; e.g., [20]) or the ensemble Kalman filter (EnKF; e.g., [3]).

The EnKF is a Monte-Carlo implementation of the Kalman filter algorithm and is often used to data assimilate oceanographic models (e.g., [3]). Location-error adjustments to the Kalman filter detailed here will also apply to the EnKF in a simple and straightforward way. In the EnKF algorithm, at each step, one needs to simulate observations 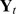, by adding 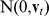 noise, (recalling that 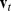 is the observation error) to build an ensemble of model predictions. As before, including location error inflates the variance of 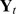. Hence, as demonstrated above, the correct variance when replicating 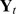 is not 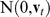 random errors but 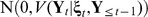 random errors, where 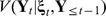 is given by (8).

Throughout this article we assumed that both the system equation and the observation equation are linear in 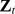
's. However, in many applications this is not the case, and the EKF was developed to tackle this particular problem. In the EKF, a linearized approximation, defined by the Jacobian, or the linear tangent operator, is used for the prediction of the error statistics. The algorithm is otherwise quite similar to the simple Kalman filter algorithm. Therefore, the adjustment for this case will be along the same lines as the corrections proposed above.

In this article, we have assumed that the process variable 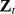 is independent and has a diagonal covariance matrix 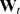. This is a simplifying assumption that enables the Kalman filter to be fitted with relative ease. However, process variables are often spatially correlated at any given time step. This correlation could be incorporated into our altered filtering scheme by specifying the covariance matrix 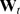 to be an appropriate, non-diagonal, structure. The results obtained in this article will still hold with this alteration.

Another simplifying assumption that we made in this article is the independence of the location errors. However, in many operational situations, location errors might not be independent over time, instead they might be auto-correlated. The adjustments to the Kalman filter based DA scheme may need further refinement from those described here, depending on the precise factor giving rise to auto-correlated location error.

In this article we consider data assimilation schemes that use Kalman filter based methods. However, these methods are not the only ones used in practice. A notable alternative are the class of variational methods (e.g., [2]). In variational methods the location of the observations are still required. A description of how this extra uncertainty should be incorporated into a variational method remains a topic of future research.

The simulation study illustrated in this article showed that the performance of the adjusted Kalman filter updates is better than the unadjusted Kalman filter updates for data prone to location error. This study has charted a way forward to address this problem. However, the methods shown here require further development and expansion into a real ocean data assimilation scheme, in order to assess the performance in the context of more complicated oceanographic models.

## Supporting Information

Appendix S1 **Glossary of notations.**
(PDF)

Appendix S2 **First-order corrections to the Kalman filter algorithm.**
(PDF)

Appendix S3 **Second-order correction to the posterior expectation.**
(PDF)
