# Supplementary material for: Accounting for Location Error in Kalman Filters: Integrating Animal Borne Sensor Data into Assimilation Schemes
Source: PLoS One. 2012 Aug 10;7(8):e42093. doi: 10.1371/journal.pone.0042093 (PMC3416853; doi:10.1371/journal.pone.0042093)
Supplement: Appendix S1 — Glossary of notations. (PDF) [file pone.0042093.s001.pdf]

Accounting for Location Error in Kalman Filters: Integrating Animal Borne Sensor Data into Assimilation Schemes

Aritra Sengupta, Scott D. Foster, Toby A. Patterson, Mark Bravington

Appendix S1: Glossary of Notation

A glossary of the notation used in this paper (taken from [1]) and that used in [2] is now given. See Table 1. It serves as a dictionary between the two sets of notations.

Table 1. The notations used in this paper, along with an explanation and the corresponding "unified" notation as defined in [2].

Our Notation	Definition in this paper	Notation as in [2]	Definition as in [2]
$\mathbf{Y}_t$	Observations at time t.	$\mathbf{y}_t^o$	Observations at time t.
$\mathbf{X}_t$	Matrix where each row corresponds to the true (unknown) location for the corresponding row of the observation vector $\mathbf{Y}_t$.	N/A	N/A
$\boldsymbol{\xi}_t$	Matrix where each row corresponds to the estimated (noisy) location for the corresponding row of the observation vector $\mathbf{Y}_t$.	N/A	N/A
$\mathbf{Z_t}$	Process variable at time t.	$\mathbf{x}(t)$	State vector at time t.
$\mathbf{F}_t(\mathbf{X}_t)\mathbf{Z}_t + \mathbf{d}_t$	Conditional mean of the observation $\mathbf{Y}_t$, conditional on $\mathbf{Z_t}$, and $\mathbf{X}_t$.	$\mathbf{H}_t\mathbf{x}(t)$	Linearized observation operator at time t.
$\mathbf{v}_t \sim \mathrm{N}(\mathbf{0}, \mathbf{V}_t)$	Observation error.	$\boldsymbol{\varepsilon}_t \sim \mathrm{N}(\mathbf{0}, \mathbf{R}_t)$	Observational error.
$\mathbf{G}_t\mathbf{Z}_{t-1} + \mathbf{c}_t$	Model defining the evolution of the mean for the process variable at time t, given information upto time point $t-1$.	$\mathbf{M}_t\mathbf{x}(t-1)$	Linearized dynamics operator at time t.
$\mathbf{w}_t \sim \mathrm{N}(\mathbf{0}, \mathbf{W}_t)$	System equation error	$\boldsymbol{\eta}(t) \sim \mathrm{N}(\mathbf{0}, \mathbf{Q}_t)$	Model error ("system noise").

References

1. Meinhold J, Singpurwalla ND (1983) Understanding the Kalman Filter. The American Statistician 37: 123-127.

2. Ide K, Courtier P, Ghil M, Lorenc AC (1997) Unified notation for data assimilation: Operational, sequential and variational. Journal of the Meteorological Society of Japan 75: 181-189.
