# Supplementary material for: Accounting for Location Error in Kalman Filters: Integrating Animal Borne Sensor Data into Assimilation Schemes
Source: PLoS One. 2012 Aug 10;7(8):e42093. doi: 10.1371/journal.pone.0042093 (PMC3416853; doi:10.1371/journal.pone.0042093)
Supplement: Appendix S2 — First-order corrections to the Kalman filter algorithm. (PDF) [file pone.0042093.s002.pdf]

Accounting for Location Error in Kalman Filters: Integrating Animal Borne Sensor Data into Assimilation Schemes

Aritra Sengupta, Scott D. Foster, Toby A. Patterson, Mark Bravington

Appendix S2: First-Order Corrections to the Kalman Filter Algorithm

Details of the first-order corrections are shown here. First we work out the expression for the expectation.

$$
\begin{aligned}
E(\mathbf{Y}_t|\boldsymbol{\xi}_t,\mathbf{Y}_{\le t-1}) &= E_{\mathbf{X}_t|\boldsymbol{\xi}_t}\left\{E\left(\mathbf{Y}_t|\mathbf{X}_t,\mathbf{Y}_{\le t-1}\right)\right\} \\
&\approx E_{\mathbf{X}_t|\boldsymbol{\xi}_t}\left\{E\left(\mathbf{Y}_t|\mathbf{X}_t=\boldsymbol{\xi}_t,\mathbf{Y}_{\le t-1}\right)+(\mathbf{X}_t-\boldsymbol{\xi}_t)^\top\left[\frac{\partial}{\partial\mathbf{X}_t}\left(E\left(\mathbf{Y}_t|\mathbf{X}_t,\mathbf{Y}_{\le t-1}\right)\right)\right]_{\mathbf{X}_t=\boldsymbol{\xi}_t}\right\} \\
&= E\left(\mathbf{Y}_t|\mathbf{X}_t=\boldsymbol{\xi}_t,\mathbf{Y}_{\le t-1}\right). \qquad (1)
\end{aligned}
$$

In the presence of location error, the expression for variance, up to the first-order, will be:

$$
\begin{aligned}
V[\mathbf{Y}_t|\boldsymbol{\xi}_t,\mathbf{Y}_{\le t-1}] &= E_{\mathbf{X}_t|\boldsymbol{\xi}_t}\left\{V\left(\mathbf{Y}_t|\mathbf{X}_t,\mathbf{Y}_{\le t-1}\right)\right\}+V_{\mathbf{X}_t|\boldsymbol{\xi}_t}\left\{E\left(\mathbf{Y}_t|\mathbf{X}_t,\mathbf{Y}_{\le t-1}\right)\right\} \\
&\approx V\left(\mathbf{Y}_t|\mathbf{X}_t=\boldsymbol{\xi}_t,\mathbf{Y}_{\le t-1}\right)+V_{\mathbf{X}_t|\boldsymbol{\xi}_t}\left\{E\left(\mathbf{Y}_t|\mathbf{X}_t=\boldsymbol{\xi}_t,\mathbf{Y}_{\le t-1}\right)+(\mathbf{X}_t-\boldsymbol{\xi}_t)^\top\left[\frac{\partial}{\partial\mathbf{X}_t}\left(E\left(\mathbf{Y}_t|\mathbf{X}_t,\mathbf{Y}_{\le t-1}\right)\right)\right]_{\mathbf{X}_t=\boldsymbol{\xi}_t}\right\} \\
&\approx V\left(\mathbf{Y}_t|\mathbf{X}_t=\boldsymbol{\xi}_t,\mathbf{Y}_{\le t-1}\right)+\left\{\left[\frac{\partial}{\partial\mathbf{X}_t}\left(E\left(\mathbf{Y}_t|\mathbf{X}_t,\mathbf{Y}_{\le t-1}\right)\right)\right]_{\mathbf{X}_t=\boldsymbol{\xi}_t}^\top V(\mathbf{X}_t|\boldsymbol{\xi}_t)\left[\frac{\partial}{\partial\mathbf{X}_t}\left(E\left(\mathbf{Y}_t|\mathbf{X}_t,\mathbf{Y}_{\le t-1}\right)\right)\right]_{\mathbf{X}_t=\boldsymbol{\xi}_t}\right\}. \qquad (2)
\end{aligned}
$$

Next we derive the first-order corrections to the covariance term in the joint distribution $[\mathbf{Z}_t,\mathbf{Y}_t]$, conditional on $\mathbf{Y}_{\le t-1}$ and $\mathbf{X}_{\le t}$:

$$
\begin{aligned}
\mathrm{Cov}(\mathbf{Y}_t,\mathbf{Z}_t|\mathbf{Y}_{\le t-1},\boldsymbol{\xi}_t) &= E_{\mathbf{X}_t|\boldsymbol{\xi}_t}\mathrm{Cov}(\mathbf{Y}_t,\mathbf{Z}_t|\mathbf{Y}_{\le t-1},\mathbf{X}_t)+\mathrm{Cov}_{\mathbf{X}_t|\boldsymbol{\xi}_t}\left(E(\mathbf{Y}_t|\mathbf{X}_t),E(\mathbf{Z}_t|\mathbf{X}_t)\right) \\
&= E_{\mathbf{X}_t|\boldsymbol{\xi}_t}\left\{\mathrm{Cov}(\mathbf{Y}_t,\mathbf{Z}_t|\mathbf{Y}_{\le t-1},\mathbf{X}_t)\right\} \\
&= E_{\mathbf{X}_t|\boldsymbol{\xi}_t}\left\{\mathrm{Cov}(\mathbf{Y}_t,\mathbf{Z}_t|\mathbf{Y}_{\le t-1},\mathbf{X}_t=\boldsymbol{\xi}_t)+(\mathbf{X}_t-\boldsymbol{\xi}_t)^\top\left[\frac{\partial}{\partial\mathbf{X}_t}\mathrm{Cov}\left(\mathbf{Y}_t,\mathbf{Z}_t|\mathbf{X}_t,\mathbf{Y}_{\le t-1}\right)\right]_{\mathbf{X}_t=\boldsymbol{\xi}_t}\right\} \\
&\approx \mathrm{Cov}(\mathbf{Y}_t,\mathbf{Z}_t|\mathbf{Y}_{\le t-1},\mathbf{X}_t=\boldsymbol{\xi}_t) \\
&= \mathbf{R}_t\mathbf{F}_t(\boldsymbol{\xi}_t). \qquad (3)
\end{aligned}
$$
