# Supplementary material for: Accounting for Location Error in Kalman Filters: Integrating Animal Borne Sensor Data into Assimilation Schemes
Source: PLoS One. 2012 Aug 10;7(8):e42093. doi: 10.1371/journal.pone.0042093 (PMC3416853; doi:10.1371/journal.pone.0042093)
Supplement: Appendix S3 — Second-order correction to the posterior expectation. (PDF) [file pone.0042093.s003.pdf]

Accounting for Location Error in Kalman Filters: Integrating Animal Borne Sensor Data into Assimilation Schemes

Aritra Sengupta, Scott D. Foster, Toby A. Patterson, Mark Bravington

Appendix S3: Second-Order Correction to the Posterior Expectation

Here we will derive the second-order correction to the expectation of the posterior distribution $[\mathbf{Y}_t|\boldsymbol{\xi}_t, \mathbf{Y}_{\leq t-1}]$. We have:

$$
\begin{aligned}
E(\mathbf{Y}_t|\boldsymbol{\xi}_t, \mathbf{Y}_{\leq t-1}) &= E_{\mathbf{X}_t|\boldsymbol{\xi}_t}\left\{E\left(\mathbf{Y}_t|\mathbf{X}_t, \mathbf{Y}_{\leq t-1}\right)\right\} \\
&\approx E_{\mathbf{X}_t|\boldsymbol{\xi}_t}\left\{E\left(\mathbf{Y}_t|\mathbf{X}_t = \boldsymbol{\xi}_t, \mathbf{Y}_{\leq t-1}\right) + (\mathbf{X}_t - \boldsymbol{\xi}_t)^\top \left[\frac{\partial}{\partial \mathbf{X}_t} E\left(\mathbf{Y}_t|\mathbf{X}_t, \mathbf{Y}_{\leq t-1}\right)\right]_{\mathbf{X}_t=\boldsymbol{\xi}_t}\right. \\
&\quad \left. + \frac{1}{2}(\mathbf{X}_t - \boldsymbol{\xi}_t)^\top \left[\frac{\partial^2}{\partial \mathbf{X}_t^\top \partial \mathbf{X}_t} E\left(\mathbf{Y}_t|\mathbf{X}_t, \mathbf{Y}_{\leq t-1}\right)\right]_{\mathbf{X}_t=\boldsymbol{\xi}_t} (\mathbf{X}_t - \boldsymbol{\xi}_t)\right\} \\
&= E\left(\mathbf{Y}_t|\mathbf{X}_t = \boldsymbol{\xi}_t, \mathbf{Y}_{\leq t-1}\right) + \frac{1}{2}\text{trace}\left\{V(\mathbf{X}_t|\boldsymbol{\xi}_t)\left[\frac{\partial^2}{\partial \mathbf{X}_t^\top \partial \mathbf{X}_t} E\left(\mathbf{Y}_t|\mathbf{X}_t, \mathbf{Y}_{\leq t-1}\right)\right]_{\mathbf{X}_t=\boldsymbol{\xi}_t}\right\}. \qquad (1)
\end{aligned}
$$
